# Genetic composition of captive panda population

**DOI:** 10.1186/s12863-016-0441-y

**Published:** 2016-10-03

**Authors:** Jiandong Yang, Fujun Shen, Rong Hou, Yang Da

**Affiliations:** 1 College of Animal Science and Technology, Sichuan Agricultural University, Chengdu, Sichuan 611130 China; 2 The Key Laboratory for Conservation Biology of Endangered Wildlife of Sichuan Province, Chengdu Research Base of Giant Panda Breeding, Chengdu, Sichuan 610081 China; 3 Department of Animal Science, University of Minnesota, Saint Paul, MN 55108 USA

**Keywords:** Giant panda, Genetic composition, Habitat, Captive breeding

## Abstract

**Background:**

A major function of the captive panda population is to preserve the genetic diversity of wild panda populations in their natural habitats. Understanding the genetic composition of the captive panda population in terms of genetic contributions from the wild panda populations provides necessary knowledge for breeding plans to preserve the genetic diversity of the wild panda populations.

**Results:**

The genetic contributions from different wild populations to the captive panda population were highly unbalanced, with Qionglai accounting for 52.2 % of the captive panda gene pool, followed by Minshan with 21.5 %, Qinling with 10.6 %, Liangshan with 8.2 %, and Xiaoxiangling with 3.6 %, whereas Daxiangling, which had similar population size as Xiaoxiangling, had no genetic representation in the captive population. The current breeding recommendations may increase the contribution of some small wild populations at the expense of decreasing the contributions of other small wild populations, i.e., increasing the Xiaoxiangling contribution while decreasing the contribution of Liangshan, or sharply increasing the Qinling contribution while decreasing the contributions of Xiaoxiangling and Liangshan, which were two of the three smallest wild populations and were already severely under-represented in the captive population. We developed three habitat-controlled breeding plans that could increase the genetic contributions from the smallest wild populations to 6.7–11.2 % for Xiaoxiangling, 11.5–12.3 % for Liangshan and 12.9–20.0 % for Qinling among the offspring of one breeding season while reducing the risk of hidden inbreeding due to related founders from the same habitat undetectable by pedigree data.

**Conclusion:**

The three smallest wild panda populations of Daxiangling, Xiaoxiangling and Liangshan either had no representation or were severely unrepresented in the current captive panda population. By incorporating the breeding goal of increasing the genetic contributions from the smallest wild populations into breeding plans, the severely under-represented small wild populations in the current captive panda population could be increased steadily for the near future.

**Electronic supplementary material:**

The online version of this article (doi:10.1186/s12863-016-0441-y) contains supplementary material, which is available to authorized users.

## Background

The giant panda (*Ailuropoda melanoleuca*) is an endangered species threatened by its own reproductive difficulties as well as habitat loss and fragmentation. As part of the effort to preserve this endangered species, *ex situ* conservation or captive breeding is important to increase the number of pandas outside their natural environment [1, 2]. A fundamental goal of *ex situ* conservation is to maintain genetic diversity that is representative of the wild population [3]. Therefore, ancestral habitats should be sufficiently represented in the captive population.

**Fig. 1:**
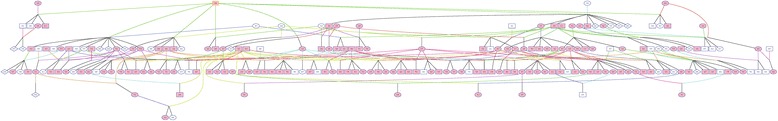
Pedigree of panda #308 with the largest genetic contribution (7.1 % of the gene pool and 125 descendants) to the captive panda population among all wild founders as of October 2014. This pedigree is an example showing the complexity of the pedigree structure of the captive panda population. The full pedigree of the captive panda population is provided as Additional file 1. Square: male. Circle: female. Diamond: sex unknown. Pink filled color: in the current population. White filled color: not in the current population. (Pedigree drawings of this figure and Additional file 1 were produced by the Pedigraph program [16])

The captive panda population has been growing steadily since 1963 when the first panda cub was born in captivity, and the number of captive-born pandas for the first time surpassed the number of wild-caught pandas in 1997. By October 2014, the worldwide captive panda population had 397 pandas and the historical panda pedigree had 944 pandas [4] with a complex pedigree structure (Fig. 1, Additional file 1). In spite of this population growth, genetic diversity of the captive population was lower than that of the wild population, indicating that the captive population only represented part of the entire gene pool of giant pandas [5]. Furthermore, the genetic contributions of wild founders were highly unbalanced with a small number of founders accounting for a large percentage of the captive gene pool [6]. The genetic composition of the captive population in terms of genetic contributions from different wild populations has not been assessed. Without addressing the issue of the genetic contributions from different wild populations, genetic diversity of small wild populations could be lost from the captive population, and the captive breeding program would not help preserve the genetic diversity from the small wild populations that need help most.

Inbreeding is typically associated with decreased fertility and survival [7] and is a threat to panda captive breeding. Current breeding recommendations focused on controlling inbreeding based on pedigree information assuming unrelated wild founders [6]. However, genetic relationships calculated using nineteen microsatellite markers revealed many potentially related pandas that had been considered unrelated by pedigree information [8]. Genomic inbreeding and relationships in wild panda populations calculated using single nucleotide polymorphism (SNP) markers from panda whole genome sequences [9] also revealed the existence of inbreeding in wild panda populations [10]. Therefore, controlling inbreeding based on pedigree information could have had hidden inbreeding due to related founders undetectable by pedigree information. Genomic coancestry coefficients between pandas from different habitats calculated using SNP markers showed that wild panda populations from the four largest habitats of Minshan, Qionglai, Qinling and Liangshan were genetically unrelated [10]. This genetic independence between the four largest wild populations provides an opportunity to use habitat-controlled breeding to avoid hidden inbreeding by using mates from different habitats.

In this study, we analyze the genetic contributions of different wild populations to the captive panda population, evaluate the expected genetic composition of the captive panda population resulting from the current breeding recommendations, and investigate the possibility to increase the genetic contributions from the smaller wild panda populations that were also under-represented in the captive population while avoiding pedigree inbreeding and hidden inbreeding.

## Methods

### Panda pedigree and breeding candidates

The historical pedigree with 944 pandas as of October 2014 [4] was used in this study. From this pedigree, we determined that 140 females and 126 males were breeding candidates for the 2016 breeding season, after removing four females and one male with unknown paternal identifications, 26 females and five males that were deemed unfit for breeding [6], and 46 males and 48 females that would be too young (<4.5 years old). With the 140 females and 126 males, 17,640 mating pairs were possible. For the hypothetical offspring of these 17,640 mating pairs, inbreeding coefficients were calculated using the MiniInbred program [11], and the results showed that 12,155 pairs were free of inbreeding and 5485 mating pairs had non-zero inbreeding coefficients. Among the 12,155 pairs free of inbreeding, 1630 pairs between 112 males and 125 females did not have founders from the same habitats and were used as the high priority mating pairs for developing habitat-controlled breeding plans.

### Calculation of founder and habitat contributions

Founder and habitat contributions were calculated through coancestry or kinship coefficients between descendants and founders in the captive panda population. Pedigree coancestry coefficients (*f*_ijk_) were calculated using the 2014 pedigree of the captive panda population [4] and the MiniInbred computer program [11]. The contribution of founder k in habitat j to the i^th^ panda (*c*_ijk_), the contribution of founder k to the captive population (*C*_k_), the contribution of habitat j to the i^th^ panda (*C*_ij_), and the contribution of habitat j to the captive population (*C*_j_) were calculated as:

1 $$ {c}_{\mathrm{ijk}}={f}_{\mathrm{ijk}}/(0.5)=2{f}_{\mathrm{ijk}} $$

2 $$ {C}_{\mathrm{k}}={\displaystyle {\sum}_{\mathrm{i}=1}^{\mathrm{h}}{\displaystyle {\sum}_{\mathrm{j}=1}^{{\mathrm{n}}_{\mathrm{j}}}{c}_{\mathrm{i}\mathrm{jk}}}}/\mathrm{n} $$

3 $$ {C}_{\mathrm{ij}}={\displaystyle {\sum}_{\mathrm{k}=1}^{{\mathrm{n}}_{\mathrm{j}}}{c}_{\mathrm{ij}\mathrm{k}}} $$

4 $$ {C}_{\mathrm{j}}={\displaystyle {\sum}_{\mathrm{i}=1}^{\mathrm{h}}{\displaystyle {\sum}_{\mathrm{k}=1}^{{\mathrm{n}}_{\mathrm{j}\mathrm{i}}}{c}_{\mathrm{i}\mathrm{jk}}}}/\mathrm{n} $$

where *f*_ijk_ = coancestry (kinship)coefficient [12] of the i^th^ descendant of founder k in habitat j, n_jk_ = number of descendants of founder k in habitat j, n_j_ = number of founders in habitat j, and n = number of pandas in the captive population. Note that the sum of all founder contributions to an individual is ${C}_{\mathrm{i}}={\displaystyle {\sum}_{\mathrm{j}=1}^{{\mathrm{n}}_{\mathrm{j}}}{\displaystyle {\sum}_{\mathrm{k}=1}^{{\mathrm{n}}_{\mathrm{j}\mathrm{i}}}{c}_{\mathrm{i}\mathrm{jk}}=1}}$ if the pedigree has no disconnected paths between the individual and its founders. However, the 2014 panda pedigree had 27 individuals with disconnected paths to their founders due to the use of mixed semen in artificial insemination, with 25 pandas each having *C*_i_ = 0.5, and 2 pandas each having *C*_i_ = 0.75_._

The mean kinship (coancestry) coefficient (MK) of individual j is defined as the average of all *f*_jk_ values between individual j and its r relatives, i.e.,

5 $$ {\overline{f}}_{\mathrm{j}}=\left({\displaystyle {\sum}_{\mathrm{k}=1}^{\mathrm{r}}{f}_{\mathrm{j}\mathrm{k}}}\right)/\mathrm{r} = \mathrm{M}\mathrm{K}\ \mathrm{of}\ \mathrm{individual}\ \mathrm{j}. $$

### Three habitat-controlled breeding plans

Three alternative plans of habitat-controlled breeding were developed to increase the genetic contributions from the smallest wild populations to the captive population while reducing the risk of hidden inbreeding due to related founders from the same habitat. Plan A sought to maximize the contributions of three under-represented habitats in the priority order of Xiaoxiangling, Liangshan, Qinling and Minshan while minimizing the contribution of Qionglai, with the restriction of ten mates per male panda. Plans B and C calculated the maximum number of mates for each male breeding candidate (m_i_) as a weighted number of mates, where the maximum number of mates allowed for a founder from each habitat is weighted by the habitat contribution from that habitat, i.e.,

6 $$ {\mathrm{m}}_{\mathrm{i}}={\displaystyle {\sum}_{\mathrm{j}=1}^{\mathrm{h}}{C}_{\mathrm{i}\mathrm{j}}{\mathrm{M}}_{\mathrm{j}}} $$

where m_i_ = maximum number of mates for the i^th^ breeding individual, *C*_ij_ = the contribution of habitat j to the i^th^ panda, and M_j_ = maximum number of mates allowed for a founder from habitat j. Equation 6 applies to both male and female breeding candidates but is mainly used for males. The M_j_ values for Xiaoxiangling, Liangshan, Qinling, Minshan, Qionglai and Sichuan were 10–8–6–2–2–1 for Plan B, and were 5–4–3–2–2–1 for Plan C. In the extreme case that the breeding candidate was a Xiaoxiangling founder, this founder would be allowed 10 mates under Plan B or 5 mates under Plan C. Similarly, a Qionglai male founder would be allowed two mates under Plans B and C. For a breeding candidate with genetic contributions from multiple habitats, the m_i_ value of Eq. 6 is an easy solution for calculating the maximum number of mates allowed per breeding candidate. Equation 6 is also a flexible solution because different M_j_ values can be used if needed. As shown in this article, many pandas already had contributions from multiple habitats. As time progresses, more captive pandas will have contributions from multiple habitats. Therefore, the formula of Eq. 6 should be increasingly useful for habitat-controlled breeding. For pairs with equal genetic composition, mating pairs with smaller MK values (${\overline{f}}_{\mathrm{j}}$ values of Eq. 5) were given higher breeding priority. Under these conditions, older breeding candidates were given higher priority than younger candidates, and mates at the same location were given higher priority than mates at different locations. For each mating pair of the three plans, we calculated the total contribution from each habitat to the hypothetical next generation using Eq. 1–4, and calculated the MK value using Eq. 5. The three habitat-controlled breeding plans were based on the 1630 pairs free of pedigree inbreeding and hidden inbreeding resulting from related founders from the same habitat. Due to the restriction on the maximum number of mates a male breeding candidate may have, some breeding pairs free of pedigree inbreeding but not free of hidden inbreeding were also used in the three habitat-controlled breeding plans.

### Analysis of genetic composition of current breeding recommendations

The annual panda breeding recommendations were based on mating suitability index (MSI) with MSI scores of 1–3 for high priority mating pairs, 4–6 for mating pairs to be avoided, and 3 F and 4 F for mating pairs involving founders with high genetic values [6]. Genetic composition for each category of mating pairs was analyzed using Eq. 1–4.

## Results

### Genetic composition of the captive panda population

**Fig. 2:**
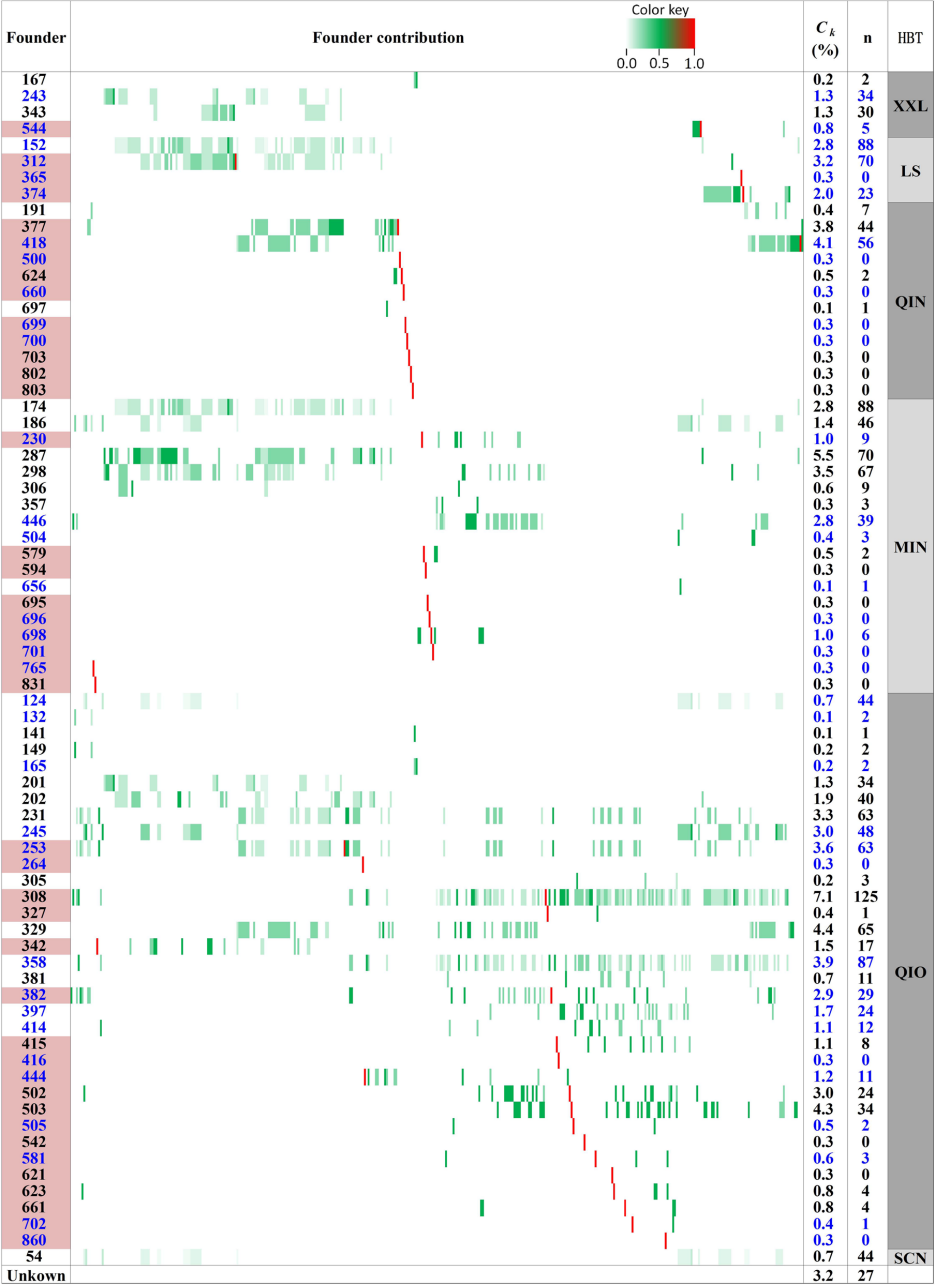
Global view of founder and habitat contributions to the captive panda population. Founder and habitat contributions were all highly unbalanced. Shaded founders were in the captive population. Blue ink indicates female founder. *C*_k_ = contribution of founder k, n = number of descendants of the founder. HBT = habitat, LS = Liangshan, MIN = Minshan, QIN = Qinling, QIO = Qionglai, XXL = Xiaoxiangling, SCN = Sichuan (could be any of Daxiangling, Liangshan, Minshan, Qionglai and Xiaoxiangling)

The analysis of the genetic contributions showed that the Qionglai (QIO) wild founders had the largest genetic contribution (52.2 %) to the captive panda population, followed by Minshan (MIN) with 21.5 %, Qinling (QIN) with 10.6 %, Liangshan with 8.2 %, Xiaoxiangling (XXL) with 3.6 %, and Sichuan with 0.7 %. The Daxiangling (DXL) population with a similar population size as Xiaoxiangling had no contribution to the captive population (Table 1, Fig. 2, Additional file 2). The only ‘Sichuan’ panda could be from any of the Qionglai, Minshan, Liangshan, Xiaoxiangling and Daxiangling populations. Unknown paternity due to mixed semen for artificial insemination accounted for 3.2 % of the genetic contributions. Approximately, the XXL-LS-QIN-MIN-QIO ratio of the genetic contributions to the captive population was 1–2.3–2.9–6.0–14.5. Translating percentages of genetic contributions to ‘full panda equivalents’ by multiplying the percentage of genetic contributions with the total number of 397 pandas yields a XXL-LS-QIN-MIN-QIO ratio of 14–33–41–85–207, i.e., the genetic contributions from Xiaoxiangling to the captive population were equivalent to fourteen Xiaoxiangling pandas, and the Liangshan contributions were equivalent to thirty three Liangshan pandas. In the wild populations, the ratio of the panda numbers was 32–115–275–708–402 (Table 1). Therefore, the fourteen ‘full pandas’ would have increased the Xiaoxiangling population by 43.8 %, a much needed help for preserving one of the most vulnerable wild panda populations. However, the two smallest wild populations with contributions to the captive panda population, Xiaoxiangling and Liangshan, were severely under-represented compared to Qionglai and Minshan. Apparently, the limited availability of mating individuals from Xiaoxiangling and Liangshan was chiefly responsible for those under-representations. The heavy use of mating individuals from Qionglai was a factor leading to the dominant representation of Qionglai, e.g., eight Qionglai founders (308, 329, 503, 358, 253, 231, 245, 502) each accounted for 3.0-7.1 % of the captive gene pool for a combined 32.6 % of the captive gene pool (Fig. 2). Clearly, breeding practice had a major impact on the genetic composition of the captive panda population.

**Table 1:** Habitat representation in captive and wild panda populations

Habitat	Founders			Wild panda population^d^	
	Number	Contribution to captive population (%)	Number of descendants	*n*	%
DXL	0	0.0	0	29	1.8
LS	3	8.2	150	115	7.2
QIN	5	10.6	113	275	17.2
MIN	12	21.5	234	708	44.4
QIO	29	52.2	347	437	27.4
SCN^a^	1	0.7	44	0	0
XXL	4	3.6	65	32	2.0
UNK^b^	-	3.2	0	0	0
Total	54	1	397^c^	1596	100

*DXL* Daxiangling, *LS* Liangshan, *MIN* Minshan, *QIN* Qinling, *QIO* Qionglai, *XXL* Xiaoxiangling, *SCN* Sichuan (could be DXL, LS, MIN, QIO or XXL)

^a^SCN was only known to be from Sichuan and could be either MIN, QIO, LS, DXL or XXL

^b^UNK = unknown habitat origin due to missing paternity resulting from the use of mixed semen in artificial insemination

^c^This is the total number of pandas in the captive population as of October 2014 and is not the summation of this column because many descendants had contributions from more than one habitat

^d^The Third National Survey of Wild Panda Population [15]

Most pandas born in captivity had genetic contributions from multiple habitats and could not be classified into any single habitat origin (Fig. 2). Moreover, the pedigree structure for inferring genetic contributions already became a complex network for many individuals (Fig. 1, Additional file 1), making the consequence of breeding plans on habitat genetic representation unpredictable unless such consequence is explicitly monitored in breeding plans as we do in this study. As time progresses, the panda’s genetic composition representing different habitats will only become more complicated. Therefore, monitoring the genetic composition of each panda in breeding plans is important for controlling the population’s habitat representation at a level most helpful for preserving the panda genetic diversity. Towards this goal in the analyses to follow, we first evaluate the genetic composition resulting from the current breeding recommendations and then explore three alternative mating plans for increasing the genetic contributions from the small wild populations while reducing the risk of hidden inbreeding.

### Expected genetic composition of captive panda population from existing panda breeding recommendations

Our analysis of the expected genetic composition resulting from the current breeding recommendations [6] showed that the recommended high priority mating pairs (MSI scores 1–3) increased the Xiaoxiangling contribution to 6.2 % from 3.6 %, reduced the Liangshan contribution to 7.6 % from 8.2 %, and had similar contributions as in the current population for Qinling (11.2 % vs. 10.6 %), Minshan (20.5 % vs. 21.5 %) and Qionglai (53.9 % vs. 52.2 %) (Table 2, Additional file 3). The increase in Xiaoxiangling contribution is desirable but the decrease in Liangshan contribution is undesirable because Liangshan was already under-represented in the current captive population. The recommended mating pairs of high genetic values (MSI scores of 3 F and 4 F) sharply increased the Qingling contribution from 10.6 % to 29.8–34.4 % while reducing the contributions from Qionglai (40.0 % vs. 52.2 %), Xiaoxiangling (0.8–2 % vs. 3.6 %) and Liangshan (3.6–4.5 % vs. 8.2 %) (Table 2). Reducing the contributions of Xiaoxiangling and Liangshan is undesirable because these two habitats were already severely under-represented in the captive population. These results showed that breeding strategy can be effective in changing the genetic contributions from the wild panda populations to the captive panda population but also showed that undesirable decreases in genetic contributions from the already less represented and smallest wild populations could occur if the resulting genetic composition of a breeding plan was not monitored and controlled.

**Table 2:** Expected genetic composition of captive panda population under the 2015 breeding recommendations

MSI	N	Habitat contribution (%)					
		XXL	LS	QIN	MIN	QIO	SCN
1	433 (247)^a^	5.8	1.4	19.9	22.7	49.9	0.3
2	1801 (1599)	6.4	8.0	11.7	19.3	53.8	0.8
3	3120 (2880)	6.2	8.2	9.7	20.9	54.1	0.9
1 + 2 + 3	5354 (4726)	6.2	7.6	11.2	20.5	53.7	0.8
3 F	1689 (846)	2.0	4.5	29.8	23.3	40.0	0.5
4 F	257 (201)	0.8	3.6	34.4	23.4	37.7	0.0
Current	397	3.6	8.2	10.6	21.5	52.2	0.7

^a^Number in () is the number of pairs with founders from the same habitat

### Expected genetic composition of captive panda population from three habitat-controlled breeding plans

We investigated three habitat-controlled breeding plans to increase the genetic contribution of the small wild populations and to reduce the risk of hidden inbreeding by incorporating parameters of habitat genetic contribution and origin into the breeding plans. Specifically, three important parameters for preserving the genetic diversity and for practical feasibility were incorporated into the three habitat-controlled breeding plans: 1) Existing genetic contribution from each wild population to a breeding candidate, 2) Expected genetic contribution from each wild population to the progeny of a mating pair, and 3) The limit number of mates a male breeding candidate may have according to the genetic contributions from different wild populations to this breeding candidate. Among the 397 pandas in the captive population, we determined that 140 females and 126 males were breeding candidates for the 2016 breeding season. From these 140 males and 126 females, 17,640 male–female pairs were possible (Additional file 4), including 12,155 pairs (68.9 %) free of pedigree inbreeding and 5485 pairs (31.1 %) with non-zero pedigree inbreeding coefficients. Among the 12,155 pairs free of pedigree inbreeding, 1630 pairs (112 males and 125 females) were free of hidden inbreeding because these pairs did not have founders with the same habitat origin (Additional file 5). These 1630 pairs were used as the high priority mating pairs in the three alternative plans of habitat-controlled breeding to increase the contributions of the less represented habitats while decreasing the contribution of Qionglai (Additional file 6). Plan A sought maximum increases in the contributions from Xiaoxiangling, Liangshan and Qinling while reducing the Qionglai contribution with the restriction of ten mates per male, while Plans B and C weighted the maximum number of mates per male for each habitat by the habitat contribution. Given the breeding goal to maximize the contributions from the small wild populations while reducing the contribution from Qionglai with the restriction on the maximum number of mates for each male mating candidate, the 1630 pairs free of hidden inbreeding could not produce all the 140 pairs needed for the 140 females. For this reason, additional pairs (30.0–67.9 % of the 140 pairs) with maximum contributions from the small wild populations and least contribution from Qionglai were selected from the 10,525 (=12,155 − 1630) pairs free of pedigree inbreeding but not free of hidden inbreeding (Table 3). Plan A used the smallest number of males and needed the smallest number of pairs with risk of hidden inbreeding, whereas Plan C used the largest number of males and needed the largest number of pairs with risk of hidden inbreeding. The three plans increased the Xiaoxiangling contribution to 6.7–11.2 %, Liangshan’s contribution to 11.5–12.3 %, Qinling’s contribution to 12.9–20.0 % and Minshan’s contribution to 23.1–27.0 %, while reducing Qionglai’s contribution to 32.8–40.3 % (Table 4). Therefore, using the three habitat-controlled breeding plans, the current XXL-LS-QIN-MIN-QIO ratio of 1–2.3–2.9–6.0–14.5 approximately could be changed to 1–2–2–3–6 in the offspring from one breeding season. These results were based on a maximum number of ten mates per breeding male. If we further allow a maximum of forty descendants per breeding male, the genetic contributions of Xiaoxiangling and Liangshan could be increased to 15 % or more in four breeding seasons based on the conservative assumption of 3 % increase in genetic contributions from Xiaoxiangling and Liangshan per breeding season.

**Table 3:** Distribution of mating pairs with and without risk of hidden inbreeding under three alternative plans of habitat-controlled breeding for 140 female breeding candidates

Plan	Number of pairs		
	Pairs from 1630 pairs free of hidden inbreeding	Pairs selected from 10,525 pairs with risk of hidden inbreeding^a^	Males
A	98	42 (30.0 %)	16
B	65	75 (53.6 %)	31
C	45	95 (67.9 %)	61

^a^These pairs were from the 10,525 pairs (=12,155 − 1630) without pedigree inbreeding but with risk of hidden inbreeding due to common habitat origin of founders

**Table 4:** Expected genetic composition of captive panda population under three alternative plans of habitat-controlled breeding for 140 mating pairs

	Habitat contribution (%)					
Plan	XXL	LS	QIN	MIN	QIO	SCN
A	11.2	11.5	20.9	23.1	32.8	0.5
B	10.6	11.9	16.3	23.8	36.9	0.4
C	6.7	12.3	12.9	27.0	40.3	0.7
Current population	3.6	8.2	10.6	21.5	52.2	0.7

## Discussion

### Power and limitation of captive breeding for increasing the genetic contributions of the smallest wild populations

The panda captive breeding program has been a great success in helping preserve this endangered species. With the current population size about 400 pandas in the captive breeding program, the captive breeding program could include a goal to increase the genetic contributions from the smallest wild populations, Xiaoxiangling, Liangshan and Qinling. The analysis of current breeding recommendations [6] and the three habitat-controlled breeding plans in this article showed that the genetic contributions from the smallest wild populations to the captive population could be increased significantly through breeding. The three habitat-controlled breeding plans can be applied routinely for panda captive breeding with updated analysis each year.

The main limiting factor for increasing the genetic contributions from the smallest wild populations is the small number of founders from the smallest wild populations of Xiaoxiangling and Liangshan, i.e., four founders from Xiaoxiangling and four founders from Liangshan (Fig. 2). Only one Xiaoxiangling founder was still in the captive population and this founder only had five descendants. Consequently, the propagation of Xiaoxiangling’s genetic contribution needed to use pandas with less than 100 % Xiaoxiangling genetics to avoid inbreeding. All four Liangshan founders were still in the captive population but two of these four founders already had seventy or more descendants, and the propagation of Liangshan’s genetic contribution also needed to use pandas with less than 100 % Liangshan genetics. The three habitat-controlled breeding plans had a restriction of ten new offspring per breeding male per breeding season to prevent creating new dominant breeding males such panda #308. Assuming this limit number for the next four years, any male breeding candidate at most could have 40 new descendants. This could be tolerable compared to the sixteen founders that each had 40–125 descendants (Fig. 2). Furthermore, a relatively large number of descendants of founders from the smallest wild populations probably should be allowed given the small number of founders available. The Qinling population was also under-represented in the captive population relative to Qionglai and Minshan, but increasing the Qinling contribution would not be as difficult as for Xiaoxiangling and Liangshan, because Qinling had five founders (Additional file 7) that could significantly increase the Qinling genetic contribution.

Introducing new male founders from Xiaoxiangling and Liangshan into the captive breeding program should be highly desirable for preserving the genetic diversity of those two wild populations through captive breeding. If opportunity exists to introduce new founders, introducing one male founder from Xiaoxiangling and one male founder from Liangshan in the next five years would be helpful for preserving the genetic diversity of these two populations.

### Needs to further evaluate panda genetic diversity in different wild populations

Xiaoxiangling is the smallest wild panda population, and introducing any new founder from Xiaoxiangling apparently is a difficult decision to make. An important question to answer is whether the Xiaoxiangling population had its unique genetic diversity that should require extra efforts to preserve. Similarly, the question whether the Daxiangling population with a similar population size as Xiaoxiangling should be represented in the captive population also needs to be answered. Unfortunately, molecular evidence to answer these questions was either limited or inconclusive.

A study using mtDNA and six MHC genes concluded that all the Sichuan derived panda populations originated from Xiaoxiangling, which had extraordinary levels of MHC diversity [13]. Such results should strongly support treating Xiaoxiangling as an independent wild panda population. However, the limited genome coverage by the mtDNA and MHC genes may not fully represent the genetic diversity of Xiaoxiangling at the genome level. Two studies using whole-genome sequences and SNP markers selected from those sequences offered whole-genome coverage but had inclusive evidence about Xiaoxiangling's genetic diversity. The sequence-based study using structure and principal component analyses classified Daxiangling, Xiaoxiangling, Liangshan and Qionglai as the same population, and classified Minshan and Qinling as two separate populations [9], whereas the analysis of genomic relationships using 150,025 SNP markers [14] selected from the same sequence data identified the only Xiaoxiangling panda to be migrated from Qionglai or to have a problem of DNA sample mixing with a Qionglai panda, identified the only Daxiangling panda to be similar to Qionglai pandas, and identified Liangshan, Qinling, Qionglai and Minshan to be four genetically independent populations [10]. The genetic diversity of Xiaoxiangling and Daxiangling should be further evaluated. Similarly, further evaluation of the Liangshan genetic diversity is needed to obtain conclusive evidence about the genetic diversity of Liangshan because only two Liangshan pandas were available in the sequence-based and SNP studies [9, 10]. Before conclusive molecular evidence becomes available, captive breeding should preserve the genetics of all wild populations because the loss of a genetic diversity is irreversible.

### Genetic composition of pandas with triplets

**Fig. 3:**
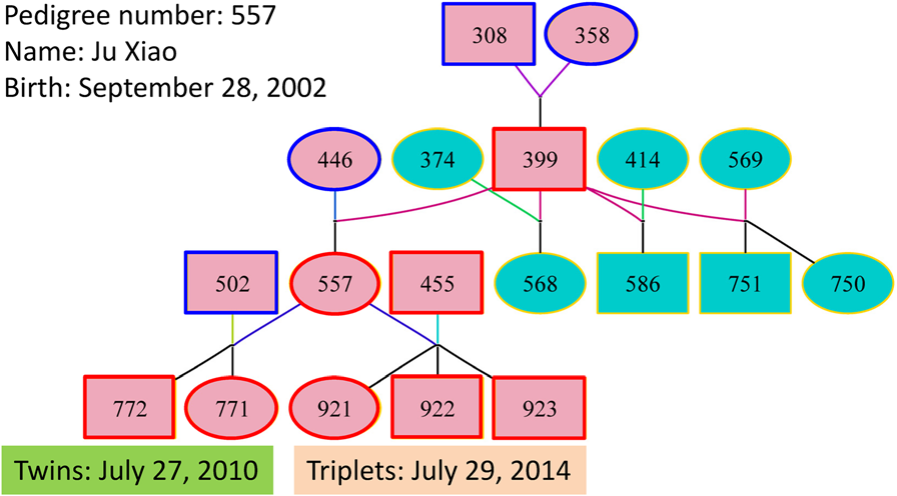
Pedigree of panda 557 giving birth to the only surviving triplets. Panda 358, grandmother of 557, also gave birth to a set of triplets on September 16 1995 with one surviving cub (434) that was still in the captive population as of October 2014. Pink fill color indicates direct family members of panda 557, and cyan fill color with yellow node color indicates pandas without genetic contribution to the cubs of panda 557. Blue node color indicates wild founders, and red node color indicates captive-born pandas in the family of panda 577

Examples of genetic composition of well-known pandas may shed light on the potential effects of habitat homogeneity and heterogeneity. We examined the genetic compositions of three female pandas giving birth to triplets. One female (358) was a Qionglai founder, while the other two females were crossbreds: panda 425 was a crossbred between Xiaoxiangling (25 %), Minshan (50 %) and Qionglai (25 %), and panda 557 with the only surviving triplets was a crossbred between Minshan and Qionglai (Table 5, Fig. 3). The fact that panda 358 was the paternal grandmother of panda 557 and both pandas 358 and 557 had triplets should be an indication of a genetic effect on the female’s ability to produce triplets. The fact that two of the three females (425 and 557) were crossbreds indicated that habitat heterogeneity did not harm the ability to produce triplets although no conclusion could be drawn whether habitat heterogeneity had any association with the ability to produce triplets.

**Table 5:** Genetic composition of three female pandas giving birth to triplets

Stud #	Institution	Parents		Habitat		
		Stud #	Sex	XXL	MIN	QIO
358	WOLONG	Wild	-	0	0	1.0
425	CHENGDU	298	M	0	1.0	0
		314	F	0.5	0	0.5
557	WOLONG	399	M	0	0	1.0
		446	F	0	1.0	0

## Conclusion

The genetic composition of the captive panda population had dominant genetic contributions from the two largest wild populations and a small fraction of the genetic contributions from the two smallest wild populations. The currently available breeding candidates would allow significant increases in the genetic contributions from the two smallest wild populations and reduce the risk of hidden inbreeding using habitat-controlled breeding plans that monitor and control the genetic contributions from different wild panda populations to the captive panda population.

## Additional files

Additional file 1: Historical pedigree of the captive panda population as of October 2014. Founders without descendants and not in the current captive population were removed from the pedigree drawing. Square: male. Circle: female. Diamond: sex unknown. Pink filled color: in the current population. White filled color: not in the current population. (PDF 156 kb) Additional file 2: Founder and habitat contributions to the captive panda population. (XLSX 100 kb) Additional file 3: Genetic composition of the new generation from the recommended mating pairs based on the MSI scores. (XLSX 3317 kb) Additional file 4: Habitat contributions and inbreeding coefficients of hypothetical offspring of all 17,640 possible mating pairs between 140 male and 126 female breeding candidates. (XLSX 5238 kb) Additional file 5: Genetic composition and inbreeding coefficients of hypothetical offspring of the 1630 mating pairs free of pedigree and hidden inbreeding. (XLSX 463 kb) Additional file 6: Genetic composition of the new generation from three plans of habitat-controlled breeding. (XLSX 58 kb) Additional file 7: Living wild founders without descendants in the captive population. (PDF 17 kb)

## Abbreviations

DXL: Daxiangling
LS: Liangshan
MIN: Minshan
MK: Mean kinship (coancestry) coefficient
MSI: Mating suitability index
QIN: Qinling
QIO: Qionglai
SNP: Single nucleotide polymorphism
XXL: Xiaoxiangling
