# Supplementary material for: Genetic composition of captive panda population
Source: BMC Genet. 2016 Oct 3;17:133. doi: 10.1186/s12863-016-0441-y (PMC5048454; doi:10.1186/s12863-016-0441-y)
Supplement: Additional file 1: — Historical pedigree of the captive panda population as of October 2014. Founders without descendants and not in the current captive population were removed from the pedigree drawing. Square: male. Circle: female. Diamond: sex unknown. Pink filled color: in the current population. White filled color: not in the current population. (PDF 156 kb) [file 12863_2016_441_MOESM1_ESM.pdf]

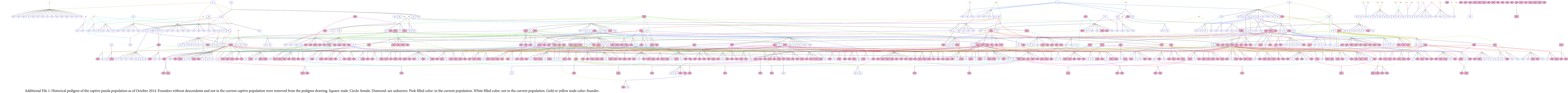

Additional File 1: Historical pedigree of the captive panda population as of October 2014. Founders without descendants and not in the current captive population were removed from the pedigree drawing. Square: male. Circle: female. Diamond: sex unknown. Pink filled color: in the current population. White filled color: not in the current population. Gold or yellow node color: founder.
