# Supplementary material for: Genetic composition of captive panda population
Source: BMC Genet. 2016 Oct 3;17:133. doi: 10.1186/s12863-016-0441-y (PMC5048454; doi:10.1186/s12863-016-0441-y)
Supplement: Additional file 7: — Living wild founders without descendants in the captive population. (PDF 17 kb) [file 12863_2016_441_MOESM7_ESM.pdf]

Additional File 7: Living wild founders without descendants in the captive population as of October 2014

Stud#	Sex	Habitat	Estimated birthdate
365	F	Liangshan	1983 ± 2yr
594	M	Minshan	1987
695	M	Minshan	1987 ± 2yr
696	F	Minshan	1995 ± 3yr
701	F	Minshan	1995 ± 3yr
765	F	Minshan	2006 ± 2yr
831	M	Minshan	2000 ± 3yr
500	F	Qinling	1985
660	F	Qinling	1997
699	F	Qinling	2006
700	F	Qinling	2006
703	M	Qinling	2007
802	M	Qinling	2009-2014
803	M	Qinling	2009-2014
264	F	Qionglai	1980
416	F	Qionglai	1993
542	M	Qionglai	1999
621	M	Qionglai	1989 ± 3yr
860	F	Qionglai	2009 ± 1yr

Shaded pandas could be too old to contribute to the captive population.
